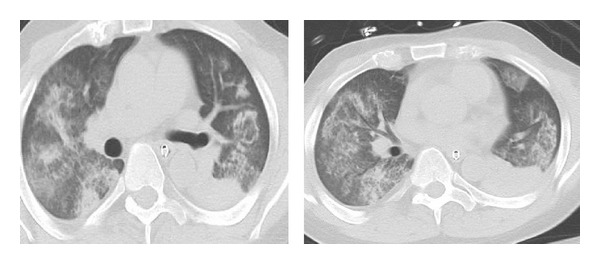# Rituximab—A Drug with Many Facets and Cures: A Treatment for Acute Refractory Hypoxemic Respiratory Failure Secondary to Severe Granulomatosis with Polyangiitis

**DOI:** 10.1155/2013/123134

**Published:** 2013-02-14

**Authors:** Braden Powers, Aditya Uppalapati, Sindhura Gogineni, Zafar Akram Jamkhana

**Affiliations:** Division of Pulmonary, Critical Care and Sleep Medicine, Saint Louis University 1402 South Grand Avenue, MC/SLUH/7 FDT, St. Louis, MO 63110-0250, USA

## Abstract

Granulomatosis with Polyangiitis (GPA) is a rare systemic anti neutrophil cytoplasmic antibody (ANCA-) associated granulomatous vasculitis of the small and medium sized blood vessels. Diffuse alveolar hemorrhage (DAH) is a rare life-threatening complication of GPA. In our patient, cyclophosphamide was held secondary to complications of acute kidney injury, hematuria, and concern for a possible hemorrhagic cystitis. However, during the workup for hematuria the patient acutely developed respiratory failure and was found to have DAH. The patient was initially supported with mechanical ventilation volume control mode, steroids, and plasma exchange. With no improvement of oxygenation, the mode of ventilation was changed to airway pressure release ventilation (APRV) and the patient was started on rituximab. The patient clinically improved over the next few days, was able to be extubated, and was transferred out of the intensive care unit.

## 1. Introduction

Granulomatosis with polyangiitis (GPA) is a necrotizing vasculitis that affects small and medium sized blood vessels with granuloma formation. It typically produces granulomatous inflammation of the upper, lower respiratory tracts and necrotizing glomerulonephritis in the kidneys. It is associated with ANCA. Its current incidence is unknown; however, its prevalence is believed to be 3/100,000 people in the USA. Diffuse alveolar hemorrhage (DAH) is identified in 25% of the patients with severe GPA [1]. We report a case of GPA with refractory hypoxemia secondary to diffuse alveolar hemorrhage that was treated with rituximab and supportive treatment.

## 2. Case Presentation

**Figure 1:**
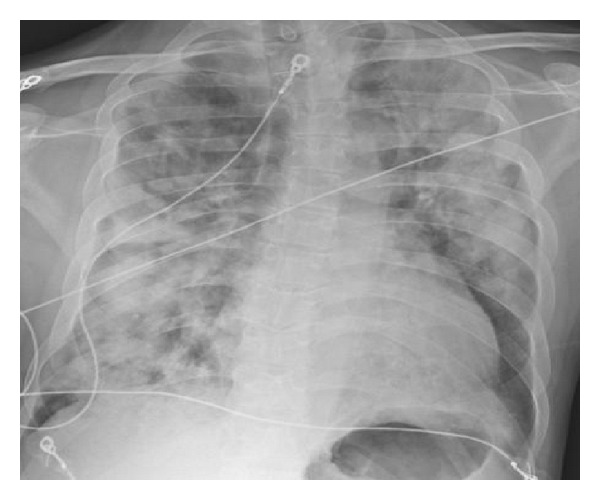
Chest X-ray showing diffuse bilateral patchy infiltrates at admission.

A 32-year-old Caucasian male with a past medical history of GPA presented with fatigue, dry cough, myalgia, and arthralgia since 2 weeks. Three days prior to presentation, he developed progressive shortness of breath. The patient did not have any hemoptysis on presentation. Previous medications included cyclophosphamide and prednisone. However, cyclophosphamide was held about a week before admission when the patient was noted to have significant hematuria, proteinuria, and possible hemorrhagic cystitis. The patient was continued on prednisone. The patient at admission was initially placed on noninvasive ventilation Bipap for acute hypoxemic respiratory failure. Vital signs at admission were as following: temperature 99.2F, heart rate 120–130 beats/min normal sinus rhythm, blood pressure 109/70 mmHg, and respiratory rate 28–34/min. Oxygen saturation was 90% on bipap (IPAP-14, EPAP-6). Chest X-ray showed bilateral diffuse patchy infiltrates (see Figure 1).

The patient's shortness of breath and mentation progressively worsened on noninvasive ventilation, and he was intubated. The patient was hypotensive after intubation and was started on norepinephrine.

**Figure 2:**
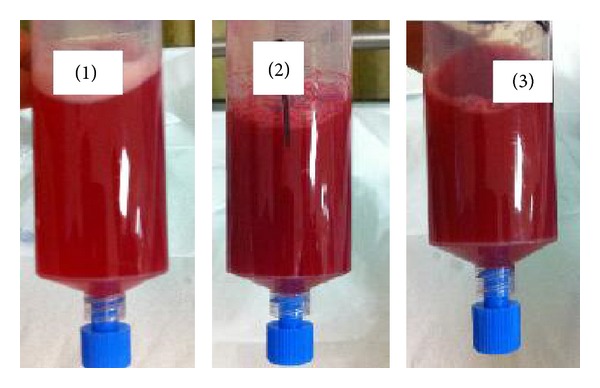
Bronchoscopy, bronchoalveolar lavage: The images show serial samples returned from bronchoalveolar lavage demonstrating progressive, hemorrhagic samples. These findings were consistent with diffuse alveolar hemorrhage.

Initial laboratory test was significant for acute rise in creatinine to 1.5 (baseline 0.9). Other relevant labs were as follows: hemoglobin is 10.0 gram/dL, hematocrit is 28.8, lactate 3.0 mmol/white blood cell count was 10.8 (10^3^/uL), INR is 1.3, and PTT is 26.4 seconds. Immunological workup showed a normal myeloperoxidase (MPO) <9.0 U/mL (reference level 0.0–9.0 U/mL), increased antiproteinase antibodies (PR3) 17.6 units/mL (reference level—0.0–3.5 U/mL), c-ANCA titer of 1 : 640, and p-ANCA <1 : 20. CRP was also elevated at 15.7 mg/dL. The patient was started on piperacillin and tazobactam, levofloxacin, vancomycin, bactrim, and micafungin. The patient had a flexible bronchoscopy that showed progressive hemorrhagic bronchoalveolar lavage in serial samples consistent with diffuse alveolar hemorrhage (see Figure 2).

The patient was then started on methylprednisolone 1 gram daily for 3 days followed by solumedrol at 1 mg/Kg in divided doses. Plasmapheresis was also started on day 1 following the diagnosis of DAH and was continued daily for 5 days. On day 2, inspite of being on a 100% FiO_2_, PEEP-18, sedated and paralyzed with cisatracurium, the patient's hypoxemia worsened and was unable to maintain PaO_2_ > 55 mmHg or SaO_2_ > 88%. Echocardiogram showed moderate pulmonary hypertension and no intra- or extracardiac shunt. The mode of ventilation was changed to APRV, and inhaled nitric oxide was also started. The patient was evaluated for extracorporeal life support (ECLS). As the patient's oxygenation started improving on APRV and nitric oxide, and the patient had bloody secretions from endotracheal tube, the patient was continued on current supportive care. He was not started on ECLS on day 4 although patient's oxygenation marginally improved with SaO_2_ ≥ 88–90%; the patient persisted to have blood secretions in the endotracheal tube with a drop in hematocrit. With all the cultures for infectious workup being negative, the patient was started on 4 doses rituximab 375 mg/m^2^ weekly. The patient was initially unstable to be moved for CT scan. However, with patient's improvement after 7 days of admission, a CT scan was obtained which showed extensive ground glass opacities, alveolar infiltrates areas of lobular consolidation, and patchy nodular foci with interstitial wall thickening compatible with GPA.

The patient clinically improved after 2 doses of rituximab and was able to be weaned off a ventilator. Hemoglobin was stable. His repeat antineutrophil cytoplasmic antibodies (c-ANCA) titer decreased to 1 : 80, antiproteinase antibodies (PR3) decreased to 8.7 U/mL, and CRP decreased to 4.0 mg/dL after therapy. The patient was able to be soon transferred out of the ICU in a stable condition. The patient received the third dose in the hospital and completed his last dose as an outpatient. The patient's repeat of PR3 further decreased to 4.0 U/mL.

## 3. Discussion

GPA is an organ and a life-threatening autoimmune disease. It was previously named after Dr. Friedrich Wegener as Wegener's granulomatosis. However, in a recent effort to have vasculitis nomenclature free of eponyms it was changed to the current name of GPA [2]. It is typically characterized by necrotizing granulomatous inflammation of the upper and lower respiratory tract, necrotizing glomerulonephritis, and an autoimmune necrotizing systemic vasculitis affecting predominantly small vessels. The American College of Rheumatology (ACR) classified GPA based on fulfilling at least 2 of 4 criteria. These criteria include the following:

- nasal or oral inflammation with development of painful or painless oral ulcers or purulent or bloody nasal discharge;
- abnormal chest radiograph showing the presence of nodules, fixed infiltrates, or cavities;
- urinary sediment characterized by microhematuria (>5 red blood cells per high-power field) or red cell casts in urine sediment;
- Granulomatous inflammation within the wall of an artery or in the perivascular or extravascular area (artery or arteriole) on biopsy [3].

The Chapel Hill Consensus (CHC) Conference defines GPA as associated with cytoplasmic pattern ANCA (c-ANCA). Both c-ANCA and PR3 antibodies are closely associated with GPA with 85–90% sensitivity and 95% specificity for generalized active disease [4].

The cause of GPA is not exactly known. GPA can occur at any age, but most often between the ages of 40 and 65. It is rare in children. The most common radiographic and CT abnormalities seen at presentation in up to 90% of patients are pulmonary nodules or consolidation with cavitation. Diffuse alveolar hemorrhage (DAH) is a less common presentation of GPA and is associated with a higher mortality [5]. 

 DAH is a clinical-pathologic syndrome characterized by disseminated injury of the pulmonary capillaries leading to accumulation of intra-alveolar RBCs originating from the alveolar capillaries. The clinical syndrome includes hemoptysis, anemia, diffuse radiographic pulmonary infiltrates, and hypoxemic respiratory failure. Hemoptysis, however, may be initially absent in up to 33% of cases. The diagnosis is established after flexible bronchoscopy shows progressively hemorrhagic BAL in serial samples [6]. In subacute or recurrent episodes, hemosiderin-laden macrophages may be useful for diagnosis. More than or equal to 20% of siderophages are considered an easier determinant of the diagnosis of DAH [7]. The chest radiograph findings are nonspecific and consist of an alveolar filling process that can be a patchy, focal, or diffuse alveolar filling process (see Figure 3).

20–40% of GPA patients appear to be cured by conventional therapy. Immunosuppressive agents are the mainstay of therapy for DAH. Cyclophosphamide and corticosteroids with subsequent tapering, according to disease activity are the mainstay for the induction treatment in patients with severe organ and life-threatening disease manifestation. Maintenance therapy after remission is achieved either with methotrexate or azathioprine. However, disease flares are common. Treatment-induced side effects are a major cause of morbidity and mortality with conventional therapy. In patients with DAH and side effects from immunosuppressive drugs, there is a limited data for supportive management. Methotrexate should not be used in the acute setting of DAH [8].

Plasma exchange has also shown usefulness if instituted early in the disease course [9]. Recently, rituximab, a chimeric anti-CD20 monoclonal antibody, has been shown to induce remission in patients with refractory ANCA-associated granulomatous vasculitis. B-lymphocytes play a predominant role in autoimmune diseases. Rituximab is a chimeric monoclonal antibody directed against CD20 that leads to selective B-lymphocyte depletion. Rituximab may be considered for refractory GPA in patients not tolerating cyclophosphamide [10, 11]. 

In patients with persistent hypoxemic respiratory failure, the patients can be managed as per ARDS net protocol. However, as our patient was persistently hypoxemic his mode of ventilator was changed to airway pressure release ventilation (APRV). APRV has shown to improve oxygenation and decreased dead space ventilation in patients with refractory ARDS and can be used as an alternative mode in patients with refractory hypoxemia on traditional volume control [12, 13]. Steroids are routinely used as a part of induction remission for GPA and also recommended for moderate and severe ARDS [14]. Based on case reports, extracorporeal life support (ECLS) can also be considered in selected patients with pulmonary hemorrhage [15–17]. The cautious use of anticoagulation should be balanced with the risk of bleeding.

Our case illustrates a successful use of rituximab in addition to the supportive treatment as an effective remission induction agent for severe GPA with DAH and refractory hypoxemic respiratory failure.

## Figures and Tables

**Figure 3:**